# Discovery of Arbuscular Mycorrhizae in Mosses of the Pottiaceae Family from the Chaco Serrano (Tucumán, Argentina)

**DOI:** 10.3390/plants14071048

**Published:** 2025-03-28

**Authors:** Myriam del V. Catania, Patricia L. Albornoz, Atilio O. Rausch, Tamara M. Ledesma, Shanshan Dong, Yuqing Cai, Yuying Zeng, Yang Liu, Guillermo M. Suárez, Javier E. Moreno

**Affiliations:** 1 Instituto Criptogámico, Sección Micología, Fundación Miguel Lillo, Miguel Lillo 251, San Miguel de Tucumán T4000JFE, Argentina; 2 Facultad de Ciencias Naturales e Instituto Miguel Lillo (UNT), Miguel Lillo 205, San Miguel de Tucumán T4000JFE, Argentina; 3 Instituto de Morfología Vegetal, Fundación Miguel Lillo, Miguel Lillo 251, San Miguel de Tucumán T4000JFE, Argentina; 4 Instituto de Agrobiotecnología del Litoral, Facultad de Bioquímica y Ciencias Biológicas, Universidad Nacional del Litoral—CONICET, Centro Científico Tecnológico CONICET Santa Fe, Colectora Ruta Nacional No. 168 km. 0, Paraje El Pozo, Santa Fe 3000, Argentina; 5 Fairy Lake Botanical Garden, Chinese Academy of Sciences, Shenzhen 518004, China; 6 State Key Laboratory of Agricultural Genomics, BGI-Shenzhen, Shenzhen 518081, China; 7 Unidad Ejecutora Lillo (CONICET-Fundación Miguel Lillo), Miguel Lillo 251, San Miguel de Tucumán T4000JFE, Argentina

**Keywords:** arbuscular mycorrhizal fungi, AMF, mycorrhiza, mosses, Chaco Serrano, *Glomus* sp., *Rhizophagus irregularis*

## Abstract

Arbuscular mycorrhizal fungi (AMF) are symbiotic fungi that associate with the vast majority of terrestrial plants. Among non-vascular plants, while AMF associations are well-documented in liverworts and hornworts, there is a broad consensus that symbiotic associations do not occur in mosses. Here, we report the presence of AMF in the living material of mosses found in Chaco Serrano (Tucumán, Argentina). We found all characteristic structures of AMF when establishing an intimate connection with two moss species of Pottiaceae (Bryophyta). While *Gertrudiella uncinicoma* exhibited AMF with both Arum- and Paris-type morphologies, *Pleurochaete luteola* only displayed an Arum-type morphology. Plant tissue samples were subjected to high-throughput sequencing for AMF identification. We determined that *Rhizophagus irregularis* was a clear dominant species in both moss species, with *Glomus* sp. also being present as a less abundant element. In addition, we also reported the presence of vesicles, arbuscules, and spores adhered to the hyphae and the presence of septate endophytes. This finding expands our understanding of the interactions between AMF and non-vascular plants and prompt us to further characterize this interaction by considering the diversity of mycorrhizal associations with concurrent implications for the ecology of mosses and the functionality of the ecosystems.

## 1. Introduction

Mycorrhizas form symbiotic associations with all major land plant lineages, except for mosses [1,2]. Mycorrhiza symbiosis is increasingly recognized as having played a central role in the successful terrestrialization of land by plants [3], likely facilitating water and nutrient uptakes in exchange of carbohydrates [4]. Land plants engage in mycorrhiza-like associations with fungi from various phyla [5], with the association with arbuscular mycorrhizal fungi (AMF, Glomeromycotina) being the most common and widespread [6].

Despite the prevalence of AMF associations with land plants, there is sparse evidence of AMF infection in mosses (Bryophyta) and a consensus that mosses are not widely infected by AMF [1,7]. On the contrary, the other two bryophyte lineages, liverworts (Marchantiophyta) and hornworts (Anthocerotophyta), are mostly colonized by AMF [8]. Symbiotic associations of mycorrhizal fungi with liverworts and hornworts have been thoroughly reported [9,10,11,12,13,14]. In mosses, evidence of interaction with AMF is limited to only a few species, and this association is consistently observed on partially senescent plants [15,16,17,18,19]. Recently, Valdes et al. (2023) described intracellular hyphae, vesicles, spores, and sporocarps, presumably from AMF, associated with rhizoids and senescent moss stems [19]. However, this study did not find AMF–arbuscules that constitute the canonical structure of an intimate AMF–plant association required for a functional symbiosis [2,19,20,21]. Under controlled experimental conditions, the growth of the moss *Funaria hygrometrica* Hedw. was enhanced on soil inoculated with a combination of mycorrhizal fungi, including *Glomus* mosseae (T.H. Nicolson & Gerd.) Gerd. & Trappe, *Gigaspora gigantea* (T.H. Nicolson & Gerd.) Gerd. & Trappe, and *Gigaspora margarita* W.N. Becker & I.R. Hall [17]. Remarkably, this study revealed the presence of mycorrhizae within moss rhizoids. In addition, a field study found AMF in 24 moss species from different habitats in China, detecting intercellular and intracellular hyphae, as well as vesicles or spores [22]. However, the study could not rule out the presence of other plant roots in moss samples. Despite this well-documented absence of AMF in mosses, genome-wide studies support the conservation of multiple genes, such as DMI1, DMI3, and IPD3, and pathways, such as strigolactone [23] and lipids synthesis [24], required for mycorrhiza formation in all major land plant lineages [7].

Thus, it is widely accepted that mosses might not establish symbiotic associations with AMF, as there is no physiological evidence for nutritional interdependence or reports showing AMF symbiosis with healthy living tissues of a moss [8,25]. Here, we conducted a microscopic survey of two Pottiaceous moss species from the Chaco Serrano Forest (Tucumán, Argentina) to identify a full set of AMF-compatible structures, including arbuscules. DNA sequencing of microbial 18S rDNA genes confirmed the presence and identification of these AMF species. We also reported the presence of septate endophytes in these two mosses: *Gertrudiella uncinicoma* (Müll. Hal.) G.M. Suárez & Schiavone and *Pleurochaete luteola* (Besch.) Thér. These findings fuel the ongoing discussion around the massive absence of mycorrhizal symbiosis in the majority of moss species and raise new questions about the ecological roles of these interactions in nature.

## 2. Results

**Figure 1:**
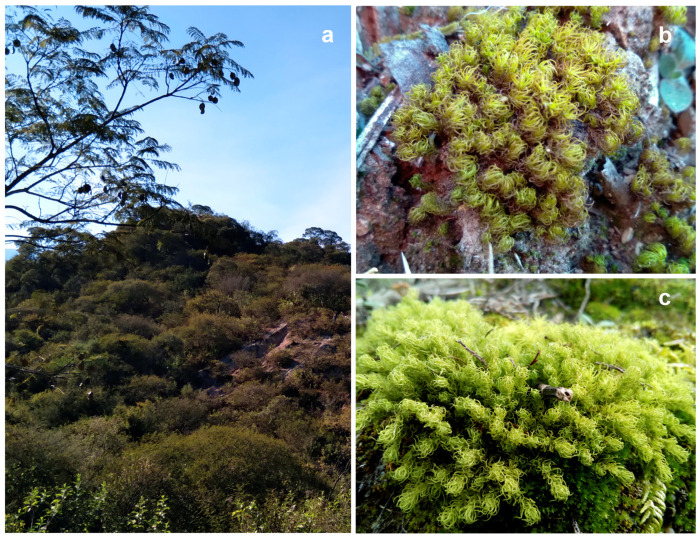
Natural habitat and general aspect of the plants. (**a**) Habitat at Chaco Serrano Forest. (**b**) *Gertrudiella uncinicoma*. (**c**) *Pleurochaete luteola*.

We searched for the presence of AMF in mosses growing in the Chaco Serrano region of Argentina, an area characterized by a subtropical biome known for its dry forests, thorny shrubs, and grasslands (Figure 1). Here, we reported the finding and identification using morphological and molecular methods of AMF on two moss species growing in this region. The moss species were *Gertrudiella uncinicoma* and *Pleurochaete luteola*, both belonging to the Pottiaceae family.

**Figure 2:**
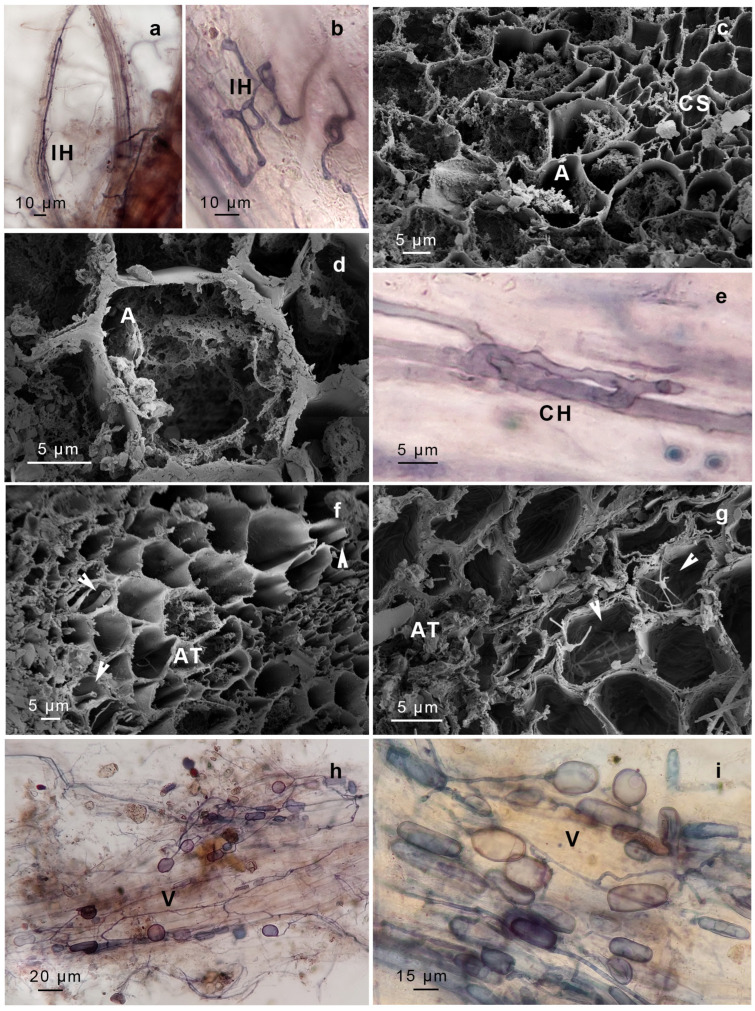
*Gertrudiella uncinicoma* colonized by arbuscular mycorrhizal fungi. (**a**,**b**) Arum-type intracellular hyphae (IH) found in leaves. (**c**,**d**) Arum-type arbuscules (A) in the stem. (**e**) Arum-type hyphae in leaves (CH). (**f**) Paris-type (arrows) morphology in stems. (**g**) Arum-type (AT) morphology in stems (arrowed). (**h**,**i**) Vesicles (V) in leaves. (**a**,**b**,**e**,**h**,**i**) Light micrographs of samples stained with trypan blue. (**c**,**d**,**f**,**g**) Scanning electron micrographs. Scale bars are in each figure.

**Figure 3:**
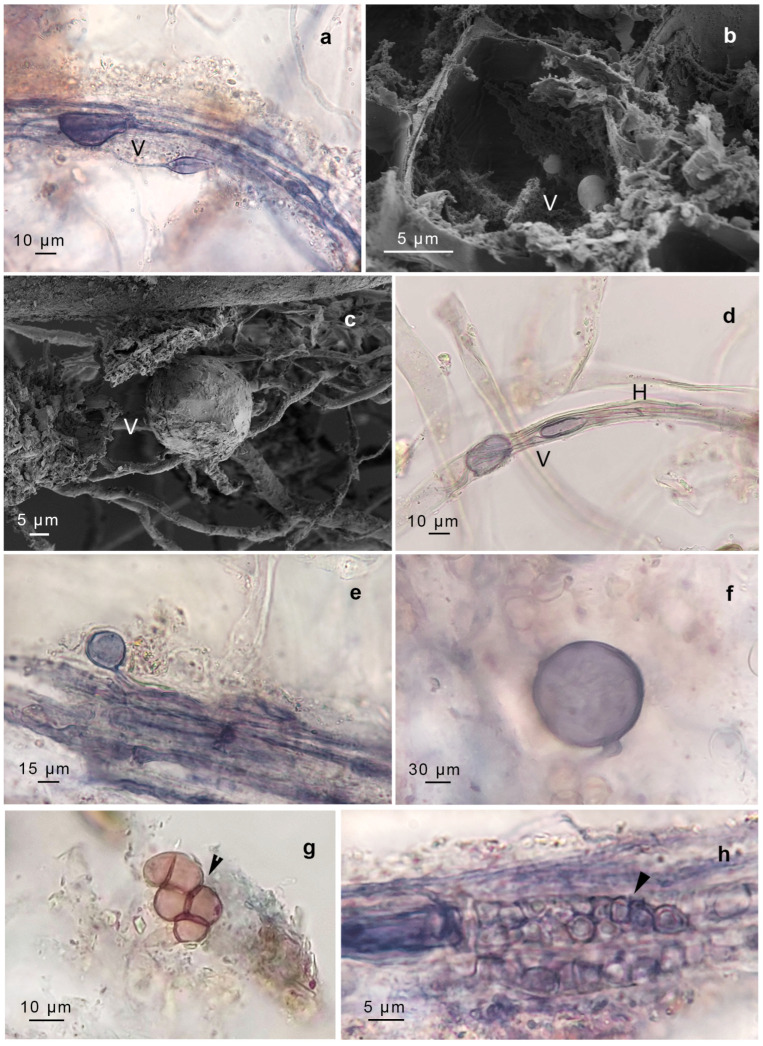
*Gertrudiella uncinicoma* colonized by arbuscular mycorrhizal fungi, and endophytic fungi. (**a**–**c**) Vesicles (V) in leaves. (**d**) Rhizoid with vesicles (V) and AMF hyphae (H). (**e**) Spores of *Glomus* sp in leaves. (**f**) Spores resembling representatives of Gigasporaceae were attached to a rhizoid. (**g**) Dark septate endophyte and microsclerotia (arrowed) in leaves. (**h**) Unknown septate fungus (arrowed) in leaves. (**a**,**d**–**h**) Light micrographs of samples stained with trypan blue. (**b**,**c**) Scanning electron micrographs. Except for panels (**d**,**f**), the rest of the observations were performed in leaves. Scale bars are in each figure.

**Figure 4:**
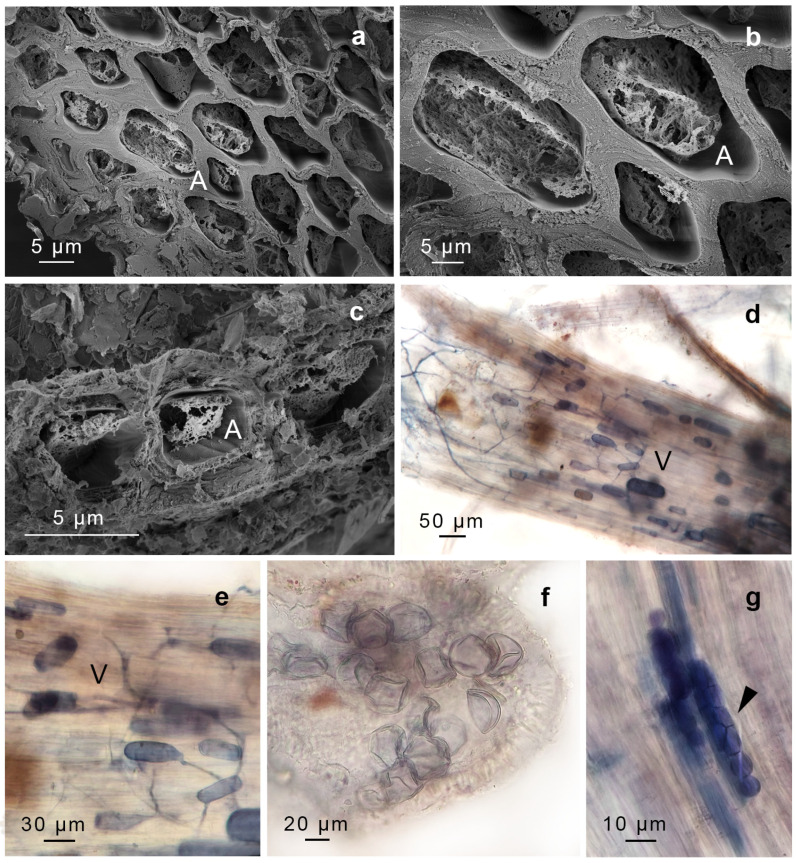
*Pleurochaete luteola* colonized by arbuscular mycorrhizal fungi and endophytic fungi. (**a**–**c**) Arbuscules (A). (**d**,**e**) Vesicle (V). (**f**) Spores of *Glomus* sp. (**g**) Unknown septate fungus (arrowed). (**d**–**g**) Light micrographs. (**a**–**c**) Scanning electron micrographs. a-g were found in stems. (**d**–**g**) are trypan blue-stained samples. Scale bars are in each figure.

We identified structures compatible with AMF, including intercellular non-septate hypha, hyphal coils (including intracellular hyphae), vesicles, arbuscules, and spores. The AMF found in *G. uncinicoma* exhibited the Arum-type morphology, characterized by the presence of both slender and broader hyphae forming an “H” shape, aligned in parallel to the surface of the gametophyte (Figure 2a,b). *G. uncinicoma* also exhibited AMF with a Paris-type morphology, displaying intracellular hyphal coils (Figure 2e–g). An Arum-type morphology with aseptate intercellular hyphae (1.5–3.0 µm wide) and arbuscules was observed in both moss species (Figure 2c,d,f,g, Figure 3a–c and Figure 4a–e).

Both species displayed ellipsoidal, globose–subglobose, and elongated vesicles connected to hyphae (Figure 2h,i, Figure 3a–d and Figure 4d,e), as well as spores probably compatible with *Glomus* sp. In addition, spores resembling representatives of Gigasporaceae were observed attached to the rhizoids of *G. uncinicoma* (Figure 3f).

In addition, two types of endophytes with intercellular hyphae and intracellular microsclerotia were observed: dark-brown septate endophytes (DSE) in leaves of *G. uncinicoma* (Figure 3g,h) and unknown septate fungi (USH) in prostrate portions of the stem of *P. luteola* (Figure 4g).

The molecular identification of AMF was carried out through the analysis of 18S rDNA sequences obtained from raw reads of *P. luteola* and *G. uncinicoma* by mapping them against a combined set of databases of 18S sequences (see Methods). To validate our bioinformatic approach, we employed published raw reads from *Marchantia paleacea* plants infected with *R. irregularis* (NCBI BioProject: PRJNA362997) [26]. As a negative control, we analyzed raw reads from *M. polymorpha*, a species that, despite its recent divergence within the *Marchantia* genus, has lost the ability to associate with AMF, a process accompanied by the pseudogenization of genes required for symbiosis, such as *CCaMK* and *CYCLOPS* [26]. As expected, our pipeline accurately detected and identified *R. irregularis* from *M. paleacea* samples, whereas it did not detect any AMF-related reads within *M. polymorpha* samples (see Methods, Table S1). Using this pipeline in both *P. luteola* and *G. uncinicoma*, we successfully identified AMF species classified under the Glomeromycotina taxon. In both mosses, among the most abundant fungi species were *R. irregularis* (Glomeraceae) and *Glomus* sp. (Glomeraceae) (Table S1). It is noteworthy that the identified *Glomus* VTX00113 was previously reported as *R. irregularis* [27], suggesting that the identified reads might actually belong to the same organism. In addition, we detected a wider range of AMF families in *P. luteola,* including Glomeraceae, Gigasporaceae, Acaulosporaceae, and Pacisporaceae, in contrast to *G. uncinicoma,* where only Glomeraceae and Gigasporaceae were detected. We applied the same approach to identify DSE and USH species. In this case, species identification was ambiguous, since we identified several candidates with similar read counts, primarily within the phylum Ascomycota and, to a lesser extent, within Basidiomycota (see Methods, Table S1).

## 3. Discussion

To fully understand the origin of mycorrhizal symbiosis in land plants, it is crucial to delve into the evolutionary history of this symbiotic association in Bryophytes, the second largest group of land plants. Fossil evidence from Rhynie Chert fossils suggests a coevolutionary history of at least ca. 410 million years between land plants and fungi of the phylum Glomeromycota [28,29]. Those fossils showed fungal structures resembling extant symbiotic associations, including vesicles, spores, intracellular coils, and arbuscule-like formations. Genomic studies further support this, revealing that genes involved in the formation of arbuscular mycorrhizal interactions are homologous to those found in other land plants and algae [7,30]. More recently, a broad presence of Mucoromycotina symbiotic fungi was also reported in all major plant lineages, including mosses [8], reopening the debate about the ancestral mycorrhizal symbionts of land plants and further reinforcing the critical role of this association for the survival and successful adaptation of early land plants during terrestrialization.

Bryophytes represent a unique group within the context of AMF–plant associations. AMF associations are well-documented in liverworts and hornworts but have been widely considered absent in mosses ([2,20,21]; among others). Previous reports described AMF growing on moss surfaces or mainly growing in senescent tissues [17,18,19], suggesting that the AMF is not establishing an intimate symbiosis with the plant but showing a behavior of an opportunistic fungus that expands within plant organs when its immune response is compromised and/or the resource allocation shifts from source to sink (typical of old senescent tissues). Interestingly, a recent work on moss-dominated biocrusts from drylands, using DNA metabarcoding and anatomical studies of a Pottiaceae moss, identified intracellular branching AMF-compatible fungi within healthy *Trichostomopsis australaceae* leaf cells [31]. Furthermore, they also found *R. irregularis* and *Glomus* sp. as the two main clades associated with moss biocrust. In our study, which is also focused on mosses from the Pottiaceae family, we reported the presence of all canonical AMF structures within the gametophytes of two healthy moss species, *G. uncinicoma* and *P. luteola*, collected from their natural habitats in the Chaco Serrano Forest (Figure 2, Figure 3 and Figure 4, Table 1). The future availability of genome sequences from members of the Pottiaceae family could help determine whether these moss–AMF interactions result from a unique evolutionary process shaping genome architecture and leading to specific morpho-anatomical adaptations that facilitate these associations or if they are primarily driven by environmental conditions that promote fungal associations. Comparative genomic analyses could reveal whether Pottiaceae mosses retain key symbiotic genes known to be essential for mutualistic interactions in vascular plants, such as *CCaMK*, *DMI3*, *RAM1*, and *CYCLOPS*, or if they have evolved novel genetic pathways to support these associations.

Our microscopic analysis confirmed AMF colonization in both moss species, with *G. uncinicoma* displaying both Arum- and Paris-type morphologies, while *P. luteola* exhibited only an Arum-type morphology. The presence of aseptate intercellular hyphae, vesicles, spores, and, most importantly, arbuscules indicates an intimate interaction between these fungi and mosses. Notably, previous studies reported *Glomus*-like structures in mosses [15,16,17,18], but none documented arbuscules—the hallmark structure of functional AMF symbiosis—within living, healthy moss gametophytes, as observed in our study. Intercellular and intracellular aseptate hyphae were evident in leaves and stems of *G. uncinicoma* (Figure 2 and Figure 3, Table 1), and they were also observed within *G. uncinicoma* rhizoids (Figure 3d, Table 1). In *P. luteola*, intercellular aseptate hyphae were only present in the stem tissues (Figure 4). In both moss species, we not only found AMF vegetative structures but also AMF arbuscules and spores thriving within the moss gametophytes. While *R. irregularis* and *Glomus* sp. were the most commonly associated fungi found in the leaf and stem of *G. uncinicoma* and in the stem of *P. luteola*, other AMF structures, including spores resembling *Gigaspora* sp., were observed at lower frequencies, primarily attached to rhizoids of *G. uncinicoma*. *Glomus* sp. has been described in mosses; however, there has been ongoing debate about whether it colonizes the interior of the stem or is restricted to its periphery [32,33,34]. Previous studies have reported *Glomus* sp. as the most commonly found AMF in mosses, primarily in the forms of hyphae and vesicles [16,17,18,19].

To complement the morphological evidence, we identified the fungal species using 18S rDNA sequences and identified AMF sequences primarily belonging to *R. irregularis* and *Glomus* sp. The molecular approach was validated using positive and negative controls, including in vitro-grown plants of *M. paleacea* (an AMF-hosting liverwort) and *M. polymorpha* (a species that lost AMF symbiosis) [26,35]. This approach validated the specificity of our detection pipeline and confirmed the absence of contaminant AMF sequences.

Although we did not directly measure fungal diversity, DNA identification provided insights into the fungal communities associated with each moss species. As mentioned before, *P. luteola* harbored a greater variety of AMF families, including Glomeraceae, Gigasporaceae, Acaulosporaceae, and Pacisporaceae, whereas *G. uncinicoma* was associated only with Glomeraceae and Gigasporaceae. This greater AMF diversity in *P. luteola* may indicate a broader range of fungal partners, suggesting a more flexible symbiotic strategy for adapting to different environmental conditions and potential differences in fungal host specificity.

In vascular plants, AMF symbiosis enhances nutrient and water uptake, particularly in resource-limited environments [36,37]. In our study, AMF-like structures were mainly present in the stems and leaves of *G. uncinicoma* and *P. luteola*. Since mosses are creeping plants whose stems and leaves can easily be in contact with the ground, one hypothesis is that the AMF-like colonization of these structures may increase water and nutrient absorption efficiency, which is crucial, given the desiccation-prone environments in which these mosses thrive.

We also reported the presence of arbuscules in the two moss species, with the co-occurrence of typical dark septate endophytes (DSE) and unknown septate fungi (USH) microsclerotia. The presence of DSE and USH alongside AMF suggests complex fungal interactions within moss tissues. These endophytes could either complement or compete with AMF, influencing nutrient dynamics and plant fitness. Future research should investigate whether these fungi facilitate AMF establishment or play independent functional roles. Interestingly, we did not find morphological or genomic evidence of Mucoromycotina in these moss species. While previous studies have reported Mucoromycotina fungi as endophytes or putative mycorrhizal partners in some bryophytes [33,38,39], their absence here could suggest species-specific differences in fungal associations, ecological constraints, or methodological limitations in detecting these fungi. Interestingly, Mucoromycotina fungi were also not reported as an endophyte of moss biocrust from drylands within healthy *Trichostomopsis australaceae,* another species of the Pottiaceae family [31]. It is also possible that environmental conditions in the Chaco Serrano Forest, as well as in the drylands, favor AMF dominance over Mucoromycotina in these mosses. Further studies incorporating targeted sequencing and broader sampling across different habitats may help clarify the potential role of Mucoromycotina in moss–fungal associations.

In conclusion, our findings enhance our understanding of the intricate relationships between AMF and non-vascular organisms, particularly the peculiar AMF–moss interaction, but also underscores the critical necessity of embracing the wide spectrum of mycorrhizal associations across diverse plant groups. Establishing in vitro cultures of AMF–moss associations will be crucial for further exploring the physiological and evolutionary implications of this newly described interaction. By illuminating the far-reaching implications for moss ecology and ecosystem functionality, this research underscores the interconnected landscape of natural systems.

## 4. Materials and Methods

Plant sampling

Moss gametophyte samples were collected from several points in the Chaco Serrano Forest from Tucumán, Argentina (Figure 1). The Chaco Serrano region in Argentina denotes a mountain transitional zone between the Yungas and the arid Chaco ecoregions. Given its transitional character, Chaco Serrano sustains a diverse ecosystem, accommodating species adapted primarily to dry and semi-dry environments. This location is made up of mountains and valleys, with plants typical of more xeric environments and with a rich floristic affinity with the most arid sectors of the Yungas. The collections were examined using traditional techniques to study the mosses, with a few modifications for Pottiaceae [40]. The methods included the examination of the internal anatomy of the leaf and stem and the use of color reactions to KOH solution. The identification of species followed current protocols [41,42].

Vouchers of the specimens were kept in the Fundación Miguel Lillo Herbarium (LIL). *G. uncinicoma* was represented by GS1912 (LIL56346), GS1955 (LIL), and GS1957 (LIL). Plants were collected in San Pedro de Colalao, Tucumán, Argentina, on 02/12/2022, with GS1912 and GS1955 being collected at 26°14′46″ S, 65°31′ W, 1125 m, and GS1957 at 26°14′02″ S, 65°30′25″ W, 1118 m. *P. luteola* was represented by GS1913 (LIL56348) and GS1956 (LIL). These specimens were also collected in San Pedro de Colalao, Tucumán, Argentina, on 2 December 2022, with GS1956 being deposited under the coordinates 26°14′02″ S, 65°30′25″ W, 1118 m.

Plants description

We presented brief comments on the morphology of the two Pottiaceae species. More detailed descriptions and illustrations were already published [40,41,42,43].

*Gertrudiella uncinicoma* (Müll. Hal.) G.M. Suárez & Schiavone

Plants formed dense cushions that were yellowish-green and turned brownish below. Stems were erect. Leaves were lanceolate, and the upper lamina was broadly channeled across the leaf; margins were strongly revolute near the apex entirely; the apex acuminated to become acute; and the base was long and elliptical (Figure 1).

*Gertrudiella uncinicoma* developed pure and dense cushions in soil in exposed places. It is a neotropical species known in Argentina, Bolivia, Paraguay, and Perú.

*Pleurochaete luteola* (Besch.) Thér.

Plants formed a deep or sprawling turf that was yellowish-green above and brown below. Stems were erect and oblong–lanceolate, and the upper lamina was broadly channeled, with the margins being a plane but occasionally recurved to become revolute along the leaf base; 1–2 marginal rows were often weakly papillose distally beyond a border of hyaline and were denticulate in the upper cells. The apex was usually sharply acute; the base was ovate to rectangular (Figure 1).

*Pleurochaete luteola* is a relatively frequent element in xerophytic deciduous forests, growing in large areas as a single species or, less commonly, mixed with other mosses. This species is distributed in North and Central America and along the Andean corridor in South America, finding its southernmost limit of distribution in northwestern Argentina.

AMF prospection in mosses

To search for stem-infecting AMF, we sampled and analyzed five individuals per species. Entire gametophytes were washed with tap water and preserved in 70% ethanol at room temperature. Then, they were clarified with 10% potassium hydroxide at room temperature for 10 to 15 days, acidified with 1% hydrochloric acid for 10 min, and stained with 0.05% trypan blue in glycerin [44]. Plant sections were placed in sterile Petri dishes and examined under a microscope to identify distinctive AMF structures. These samples were mounted in water–glycerine (1:1) to be further examined with a stereoscopic microscope (Olympus SZX7, Tokyo, Japan) or an optical microscope (Axiostar Plus, Zeiss, Göttingen, Germany) and photographed with a digital camera (Canon A620, Power Shot 7.1 MP, Tokyo, Japan).

Cytological studies using scanning electron microscopy of the AMF-infected stem

The material was further studied using scanning electron microscopy (SEM). To prepare for SEM, the plant tissue was initially fixed in Karnovsky solution [45], subjected to critical point drying, and then coated with a layer of gold–palladium using the Dorador Ion Spotter (JFC 1100 Joel, Tokyo, Japan) and observed using a scanning electron microscope (Supra 55VP, Zeiss, Oberkochen, Germany). Samples embedded in resin were sliced into 0.5 μm thick sections using a diamond histo-knife, stained with 0.5% toluidine blue, and captured through imaging using a Zeiss Axioscope light microscope (Carl Zeiss AG, Jena, Germany), which was outfitted with an MRc digital camera.

The type of colonization was based on the presence of inter- and intracellular hyphae, arbuscules, hyphal coils, and arbuscular hyphal coils [46]. The identification of AMF spores was based on morphological analyses of spore wall structures, structures of the spore bases, and subtending hyphae [34,47,48], along with the information from the International Culture Collection of Vesicular Arbuscular Mycorrhizal Fungi (https://invam.ku.edu/), accessed on 2 February 2023.

Molecular identification of AMF

DNA sequencing was performed using three independent samples of GS1956 (*P. luteola*) and GS1957 (*G. uncinicoma*). High-molecular-weight genomic DNA was prepared following well-established protocols for plant samples [49]. Libraries for short-read whole-genome sequencing (WGS) were generated utilizing the MGIEasy FS DNA Library Prep Set (Item No.1000006988), with fragment sizes ranging from 300 to 500 base pairs. Subsequently, sequencing was performed on the MGI-SEQ platform, yielding 150 base pair paired-end (PE) reads. In addition, all moss genomes were sequenced using single-tube long-fragment read (stLFR) technology to produce long-range sequences. The stLFR libraries were generated using the MGIEasy stLFR Library Prep Kit (Item No. 1000005622) [50] and subsequently sequenced on the MGI-SEQ platform. To assemble the stLFR sequences, we used the STLFR2SUPERNOVA pipeline with default parameters [51]. The assembled contigs were filtered for the non-Viridiplantae contigs that were later used to identify the taxonomic classification of fungal species using the BLASTn procedure in the NCBI, RFAM, and MaarjAM (https://maarjam.ut.ee for AMF) databases. All databases were accessed between 26 June 2023 and 1 November 2023.

Briefly, we mapped the raw reads of *P. luteola* and *G. uncinicoma* to the corresponding assembled contigs using Bowtie2 (v2.2.5) [52] to filter out non-Viridiplantae reads. For reads identified as non-Viridiplantae, we conducted a random sampling of 50% using the reformat.sh script from the BBmap package (v38.18) [53] to obtain a manageable set of reads. To perform the AMF identification, we prepared a single concatenated dataset using three public databases with nuclear ribosomal SSU (small subunit rRNA) sequences: (1) the MaarjAM database (https://maarjam.ut.ee/, updated in 2019) was prioritized due to its specialization in AMF 18S rDNA sequences, providing taxonomically curated and ecologically relevant reference sequences; (2) the NCBI SSU database (SSU eukaryote rRNA, updated in 2022) was included to broaden the reference set and account for potential non-AMF fungal species present in the samples; and (3) the RFAM database (16S rRNA, RF00177, SSU_rRNA_bacteria and 18S rRNA, RF01960, SSU_rRNA_eukarya) was used to cross-validate ribosomal RNA sequences and prevent false-positive assignments by confirming taxonomic identity at a broader phylogenetic level [54]. All datasets were concatenated to avoid potential random alignments in AMF 18S sequences. Subsequently, Bowtie2 was employed for highly sensitive local alignment (--very-sensitive-local parameter) to map non-Viridiplanteae reads against the generated database. Next, we filtered the alignment file using SAMtools (v1.9) [55], retaining only primary alignments (“-F 256” flag) with a QMAP greater than or equal to 20 (“-q 20” flag) to ensure alignment uniqueness. We employed a quality threshold (QMAP ≥ 20) to retain high-confidence reads and minimize the inclusion of erroneous sequences due to sequencing artifacts. This threshold was selected based on standard practices in high-throughput sequencing studies to balance read retention with data accuracy. Finally, the obtained results were processed using a custom R script (executed in R version 4.3.1), where tables were generated, and the number of mapped reads that passed the aforementioned filters in each case was normalized per million aligned reads against the SSU database for comparison. The outline of this pipeline is shown in in Figure S1 and was implemented for the processing of raw reads and AMF identification using the Pirayú cluster (Conicet-Santa Fe).

We validated the pipeline using published datasets from *Marchantia paleacea* infected with *R. irregularis* (NCBI BioProject: PRJNA362997) as a positive control and *Marchantia polymorpha* (NCBI BioProject: PRJNA53523) as a negative control, confirming that AMF sequences were detected only in the *M. paleacea* datasets. For the positive control, we used reported sequences from *Marchantia paleacea* plants infected with *R. irregularis* [26]. We used raw reads from a paired-end library (NCBI SRA: SRR5198766), generated with Illumina HiSeq 2500 and a 300 bp insert size as part of an *M. paleacea* sequencing project (NCBI BioProject: PRJNA362997) [26]. As a negative control, we used raw reads from *Marchantia polymorpha* L. Tak-1. Despite *M. paleacea* and *M. polymorpha* being recently diverged lineages, *M. polymorpha* lost its ability to associate with AMF, including the pseudogenization of core genes of the symbiotic response, such as *CCaMK* and *CYCLOPS* [26]. We tested our bioinformatic pipeline using raw reads from another paired-end library generated with Illumina HiSeq 2500 (NCBI SRA: SRR1800536) in the context of the *M. polymorpha* L. Tak-1 sequencing initiative (NCBI BioProject: PRJNA53523) [35]. We applied the same pipeline in both instances, with the exception of filtering TruSeq adapters and PCR primers using cutadapt (v1.18) [56], and performed quality filtering of bases using Trimmomatic (v0.39) [57]. This latter process included sliding window filtering (SLIDINGWINDOW:4:15) and minimum lengths of 90 and 215, respectively (MINLEN), as observed in the FastQC (v0.12.1) [58] quality report. Minimum read length was set at 90 bp for paired-end reads, ensuring that retained reads contained sufficient taxonomic resolution while reducing the potential for misalignments.

## Figures and Tables

**Table 1:** Comparison of morphological and molecular data for AMF identification in *P. luteola* and *G. uncinicoma*. Notes on congruence: high: strong agreement between microscopy and molecular data; moderate: partial congruence, with some structures detected morphologically but not fully resolved in sequencing data; and low: morphological structures observed, but molecular confirmation is ambiguous or incomplete.

AMF Structure Observed	Presence in *P. luteola* (GS1956)	Presence in *G. uncinicoma* (GS1957)	Molecular Identification (18S rDNA Sequencing)	Congruence
Molecular Identification (18S rDNA Sequencing)	Glomeraceae (*R. irregularis* and *Glomus* sp.), Gigasporaceae, Acaulosporaceae, Pacisporaceae, Diversisporaceae	Glomeraceae (*R. irregularis* and *Glomus* sp.), Claroideoglomeraceae	Matches detected AMF families	High (sequencing supports the identification of *R. irregularis* and *Glomus* sp.; other fungal species were not morphologically identified)
Arbuscules	Present (stems)	Present (stem, leaves)		
Intercellular aseptate hyphae	Present (stems)	Present (leaves, stems, and rhizoids)		
Intracellular hyphal coils (Paris-type morphology)	Not observed	Present (stems)		
Intracellular hyphal coils (Arum-type morphology)	Present (stems)	Present (leaves, stems)		
Vesicles	Present (stems)	Present (stems, leaves, rhizoids)		
Spores	Present (likely *Glomus* sp. in stems)	Present (rhizoids, resembling *Gigaspora* sp.)		Moderate (*Gigaspora*-like spores observed, but molecular data primarily detected Glomeraceae; *Glomus* sp. observed and supported with sequencing)
Dark Septate Endophytes (DSE)	Not observed	Present (leaves)	Ascomycota, Basidiomycota (ambiguous)	Low (morphology suggests endophytic fungi, but taxonomic assignment remains uncertain)
Unknown Septate Fungi (USH)	Present (stems)	Present (leaves)	Ascomycota, Basidiomycota (ambiguous)	Low (morphology suggests endophytic fungi, but taxonomic assignment remains uncertain)

## Data Availability

Publicly archived dataset results can be found at NCBI BioProject: PRJNA362997; NCBI SRA: SRR1800536; and NCBI BioProject: PRJNA53523.

## Supplementary Materials

The following supporting information can be downloaded at: https://www.mdpi.com/article/10.3390/plants14071048/s1, Figure S1: Outline of the bioinformatic pipeline used in this study, Table S1: Identification of AMF species using short-read sequencing. AMF species identified using the reported pipeline and filtered using q ≥ 20 for each plant species.
