# Supplementary material for: Discovery of Arbuscular Mycorrhizae in Mosses of the Pottiaceae Family from the Chaco Serrano (Tucumán, Argentina)
Source: Plants (Basel). 2025 Mar 28;14(7):1048. doi: 10.3390/plants14071048 (PMC11991092; doi:10.3390/plants14071048)
Supplement: Supplementary file 1 [file plants-14-01048-s001.zip › plants-3528971-supplementary.pdf]

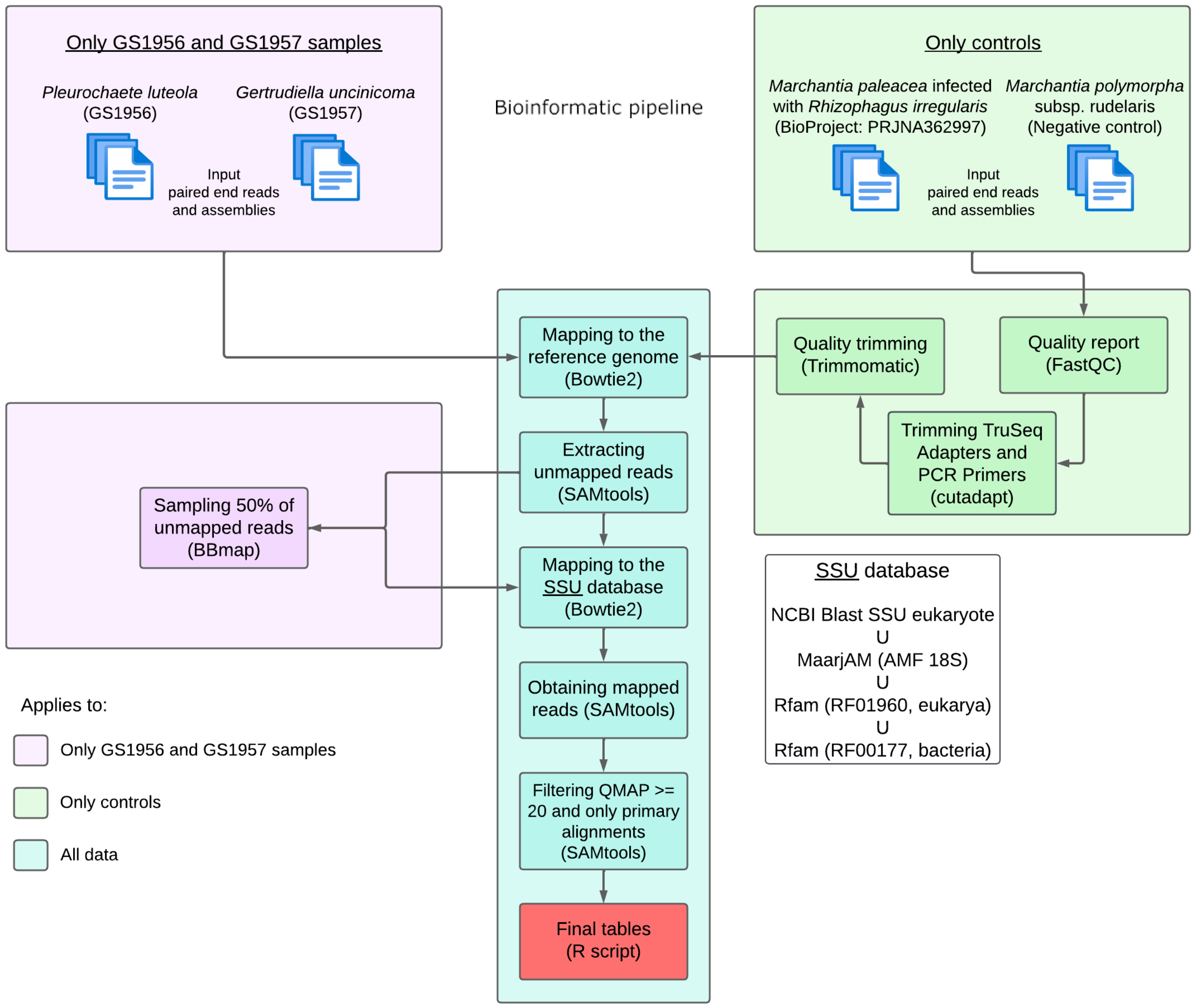

Figure S1: Outline of the bioinformatic pipeline used in this study.

This figure provides a schematic overview of the bioinformatic pipeline used for AMF identification in *Pleurochaete luteola* (GS1956) and *Gertrudiella uncinicoma* (GS1957), detailing the sequential steps from DNA extraction and sequencing to computational filtering and taxonomic classification. DNA sequencing was performed using three independent samples per species. High-molecular-weight genomic DNA was extracted, and sequenced using the MGI-SEQ platform, generating 150 bp paired-end (PE) reads, while stLFR technology was used for long-range sequencing. Then, raw reads were mapped to assembled contigs using Bowtie2 to filter out non-Viridiplantae sequences. Non-plant reads were further processed using BLASTn searches against three public databases: (1) the MaarjAM database for AMF-specific 18S rDNA sequences, (2) the NCBI SSU database for broader eukaryotic sequences, and (3) the RFAM database to cross-validate ribosomal RNA sequences and minimize false-positive assignments. The pipeline applied quality filtering, retaining only reads with a QMAP score ≥20 to ensure alignment accuracy.

To validate the pipeline, published datasets from *Marchantia paleacea* infected with *Rhizophagus irregularis* (NCBI BioProject: PRJNA362997) served as a positive control, while *Marchantia polymorpha* (NCBI BioProject: PRJNA53523), which has lost the ability to associate with AMF, was used as a negative control. The bioinformatic workflow was executed using the Pirayú cluster (CONICET-Santa Fe).

Pleurochaete luteola **GS1956 - 0.852 million of filter reads (QMAP-value >= 20, higher = more unique)**

Reads (f and R) per million	Percent of mapped reads to SSU database	Species
710.093897	0.5707%	*Rhizophagus irregularis*
223.0046948	0.0223%	Glomeraceae *Glomus* sp.
5.868544601	0.0006%	Gigasporaceae *Scutellospora* sp.
4.694835681	0.0005%	Acaulosporaceae *Acaulospora* sp.
2.34741784	0.0002%	Archaeosporaceae *Archaeospora* sp.
1.17370892	0.0001%	*Pacispora scintillans*
1.17370892	0.0001%	Diversisporaceae *Diversispora* sp.

Gertrudiella uncinicoma **GS1957 - 0.442 million of filter reads (QMAP-value >= 20, higher = more unique)**

Reads (f and R) per million	Percent of mapped reads to SSU database	Species
414.0271493	0.0413%	*Rhizophagus irregularis*
223.9819005	0.0224%	Glomeraceae *Glomus* sp.
9.049773756	0.0009%	Claroideoglomeraceae *Claroideoglomus* sp.

Marchantia paleacea **- 0.037 million of filter reads (QMAP-value >= 20, higher = more unique)**

Reads (f and R) per million	Percent of mapped reads to SSU database	Species
135.1351351	0.01%	Glomeraceae *Glomus* sp.
81.08108108	0.01%	*Rhizophagus irregularis*

Marchantia polymorpha subsp. ruderalis **- 1.032 million of filter reads (QMAP-value >= 20, higher = more unique)**

No reads mapping with QMAP-value >=20 (EMPTY)

Table S1: AMF species identified using our bioinformatic pipeline and filtered using q>=20 for each plant species.
